# Transcriptome Analysis Revealed GhWOX4 Intercedes Myriad Regulatory Pathways to Modulate Drought Tolerance and Vascular Growth in Cotton

**DOI:** 10.3390/ijms22020898

**Published:** 2021-01-18

**Authors:** Muhammad Sajjad, Xi Wei, Lisen Liu, Fuguang Li, Xiaoyang Ge

**Affiliations:** 1 State Key Laboratory of Cotton Biology, Institute of Cotton Research, Chinese Academy of Agricultural Sciences, Anyang 455000, China; muhammadsajjad.pbg@hotmail.com (M.S.); liulisen2012@163.com (L.L.); 2 Institute of Cotton Research, Henan Normal University Research Base of State Key Laboratory of Cotton Biology, Xinxiang 453000, China; chinaweixi521@163.com

**Keywords:** transcription factor, RNA-seq, vasculature, transport, knock-down, promotor zone

## Abstract

Cotton is a paramount cash crop around the globe. Among all abiotic stresses, drought is a leading cause of cotton growth and yield loss. However, the molecular link between drought stress and vascular growth and development is relatively uncharted. Here, we validated a crucial role of GhWOX4, a transcription factor, modulating drought stress with that of vasculature growth in cotton. Knock-down of GhWOX4 decreased the stem width and severely compromised vascular growth and drought tolerance. Conversely, ectopic expression of GhWOX4 in *Arabidopsis* enhanced the tolerance to drought stress. Comparative RNAseq analysis revealed auxin responsive protein (AUX/IAA), abscisic acid (ABA), and ethylene were significantly induced. Additionally, MYC-bHLH, WRKY, MYB, homeodomain, and heat-shock transcription factors (HSF) were differentially expressed in control plants as compared to GhWOX4-silenced plants. The promotor zone of GhWOX4 was found congested with plant growth, light, and stress response related cis-elements. differentially expressed genes (DEGs) related to stress, water deprivation, and desiccation response were repressed in drought treated GhWOX4-virus-induced gene silencing (VIGS) plants as compared to control. Gene ontology (GO) functions related to cell proliferation, light response, fluid transport, and flavonoid biosynthesis were over-induced in TRV: 156-0 h/TRV: 156-1 h (control) in comparison to TRV: VIGS-0 h/TRV: VIGS-1 h (GhWOX4-silenced) plants. This study improves our context for elucidating the pivotal role of GhWOX4 transcription factors (TF), which mediates drought tolerance, plays a decisive role in plant growth and development, and is likely involved in different regulatory pathways in cotton.

## 1. Introduction

Cotton (*Gossypium* spp.) is an imperative crop around the world, which offers fiber, oil, and biofuel [1]. During the life cycle, cotton is exposed to various abiotic stresses, including drought, heat, salt stress, etc. [2]. Due to the global climate change, drought is becoming the chief limiting factor that adversely affects cotton growth and productivity [3]. There is an immense need to find drought resistant germplasm and to comprehend biochemical and genetic mechanisms in plants. By developing drought resistant plants, cotton growth and productivity can be improved to meet the future requirements [4]. To muddle through these challenges, plants have evolved a series of mechanisms to perceive and respond to the diversified environmental effects through signaling pathways with apt physiological and biochemical modifications [5]. The instant response to drought stress might be turgor pressure by ion and water transport systems across cells, which induce water prevention through stomatal closure [6]. Different genes induce in plants that directly defend from stress, including transcription factors (TFs) that regulate gene expression and signaling pathways (phytohormones) to respond to the stress [7]. Plants produce anti-oxidant species such as peroxidases, proline, catalases, and superoxide dismutase etc. [2].

Plants also have developed a sophisticated vascular system that spreads in the plant body to act as a conduit for nutrients and water as well as to dispense physical strength. Vasculature mainly comprises cambium, xylem, and phloem [8]. Cambium adds up the cells to produce secondary xylem and phloem, leading to an increase in stem width. In vascular transportation, xylem conducts water and nutrients, and phloem conducts the photosynthetic products to different parts of the plant [8]. Turgor pressure and ATP are needed to carry out this active vascular transport in plants. Through systematic, long-distance signaling, vascular tissues are inter-connected. Intriguingly, sap of the vascular tissues transports micro-molecules such as sugars, metabolite, and phyto-hormones, along with macro-molecules, e.g., proteins and RNA species [9]. Notably, plants use vasculature not only to transport indispensable substances but also as a long-distance communication (LSD) route to adapt to the environmental changes at the whole plant level [10].

Previous studies have reported that WUSCHEL-related homeobox 4 (WOX4) is preferentially expressed in cambia [11] promoting and maintaining vascular cambium [12]. In *Arabidopsis*, AtWOX4 is involved in vascular cell proliferation by regulating vascular cell division [13]. In rice, OsWOX4 is involved in regulating multiple genes, including control cell progression and phyto-hormone signaling in developing vascular bundles [14]. In poplar, PttWOX4 has a crucial role in cambia and severely affects plant growth and stem girth by regulating the cell division in vascular tissues [15,16].

Increasing numbers of studies have focused on the roles of the WOX gene family in abiotic stresses. OsWOX11 mediates the ERF/WOX11 complex, modulating root growth, which leads to drought tolerance [17]. OsWOX13 differentially regulates in vascular tissues and is highly induced in response to dehydration [18]. Little is known about the role of WOXs in response to abiotic stresses in cotton. To date, there is no work reported to our knowledge explicitly depicting the regulatory network of GhWOX4-mediating drought stress response or which molecular cross-talk takes place when GhWOX4 regulates vasculature growth with that of drought stress in cotton. A potential role of GhWOX4 in regulating plant tolerance to drought stress was studied by overexpression in *Arabidopsis* and by virus-induced gene silencing (VIGS) in (*Gossypium hirsutum*). We conducted transcriptomic study to identify important differentially expressed genes (DEGs), while gene ontology (GO) enrichment revealed biological pathways differentially induced in TRV:156-0 h/TRV: 156-1 h (control) as compared to TRV: VIGS-0 h/TRV: VIGS-1 h (GhWOX4-silenced) plants. Furthermore, anatomical study and in silico promotor analysis were performed to thoroughly explore the molecular mechanisms underlying the GhWOX4-mediated cotton defense response under drought stress and growth check. Our study reports that GhWOX4 is a key gene that mediates drought tolerance and vascular growth via modulating the complex and interconnected network of transcriptional activity and signaling pathways that involve key phytohormones, TFs, and target genes. This study provides new insights into how drought tolerance, plant development, and growth might be interlinked through a master regulator—GhWOX4—in cotton.

## 2. Results

### 2.1. Identification and Structure Analysis of GhWOX4

**Figure 1:**
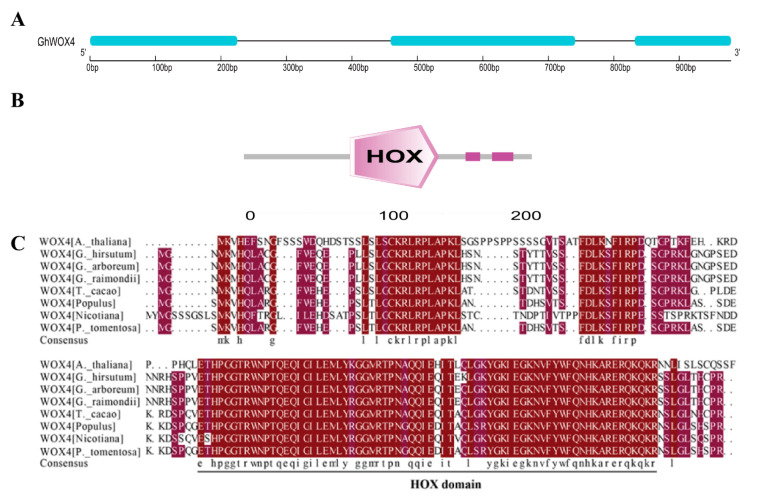
GhWOX4 domain and sequence alignment. (**A**) Gene structure: solid blue lines indicate exons, and black lines indicate introns. (**B**) Protein domain. (**C**) Protein sequence comparison of different plant species, *Arabidopsis thaliana*, *Gossypium hirsutum*, *Gossypium arboreum*, *Gossypium raimondii*, *Theobroma cacao*, *Populus trichocarpa*, *Nicotiana benthamiana* and *Populus tomentosa*.

On the basis of unpublished RNA-Seq data of *Gossypium hirsutum* L. under drought treatment, we identified a WOX gene that had early response and was significantly upregulated under drought treatment (Supplementary Figure S1). Three exons containing coding sequence (CDS) of GhWOX4 was cloned (Figure 1A). Protein sequence analysis with different plant species bared that GhWOX4 had one typical HOX domain, akin to most other members of the WOX gene family (Figure 1B,C). BLASTP analysis proved this WOX gene had a high degree of sequence similarity with AtWOX4, and it was therefore keyed out as GhWOX4 (GhWOX4a-At) (Figure 1C).

### 2.2. Sub-Cellular Localization and Phylogenetic Analysis of GhWOX4

**Figure 2:**
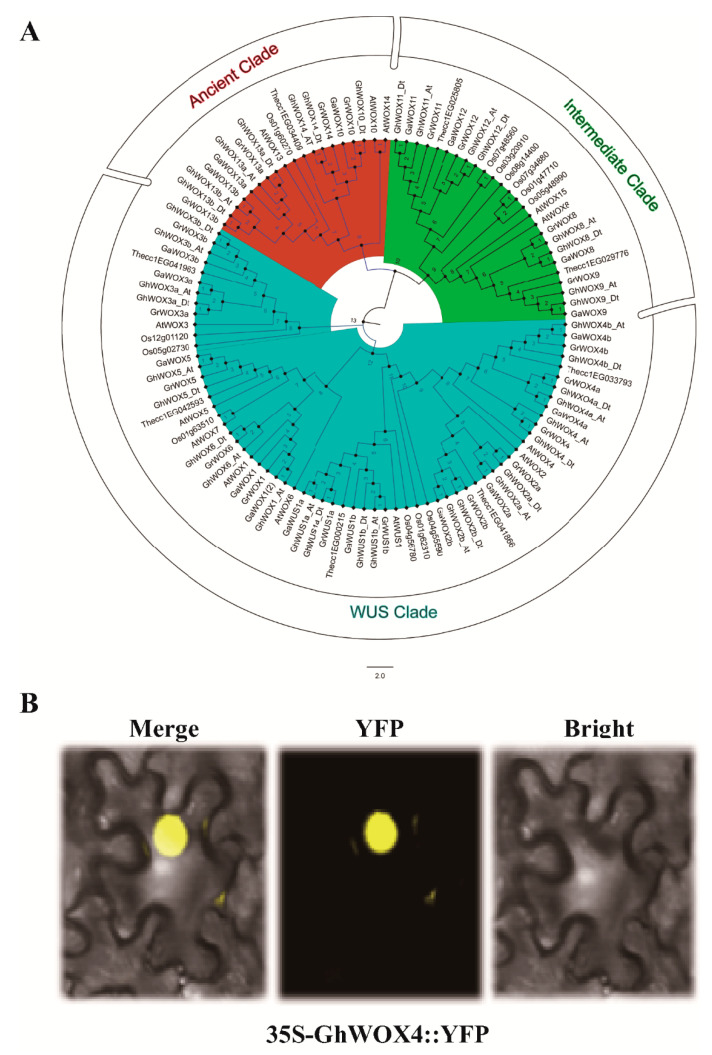
Phylogenetic analysis and subcellular localization. (**A**) Phylogenetic tree representing evolutionary relationship among WUSCHEL-related homeobox (WOX) family genes of different plant species. ClustalX was used to align protein sequences, and Mega-X with neighbor-joining method was used to construct phylogenetic tree. (**B**) Subcellular localization signal of GhWOX4, (YFP) yellow fluorescent protein.

A phylogenetic tree with different plant species was constructed to verify gene clade and similarity. To reveal the evolutionary pattern and the phylogenetic relationships of WOX genes, a phylogenetic tree was constructed using the protein sequences of WOXs from *Gossypium hirsutum* and five other plant species (*Gossypium arborium*, *Gossypium raimondii*, *Theobroma cacao*, *Oryza sativa,* and *Arabidopsis thaliana*). The result showed that the WOX gene family was classified into three types of clades: ancient clade, intermediate clade, and wus-clade. GhWOX4 was present in the biggest wus-clade of the WOX gene family and had a closed evolutionary relationship with AtWOX4 (Figure 2A).

To predict the sub-cellular localization, GhWOX4-YFP expression vector was constructed [19]. Similar to most other WOXs, GhWOX4 was located in the nucleus of the cell. Localization in the nucleus led us to know this protein may work as a transcription factor (Figure 2B), as reported in previous studies [11,13,20].

### 2.3. Overexpression of GhWOX4 Enhanced Drought Tolerance

**Figure 3:**
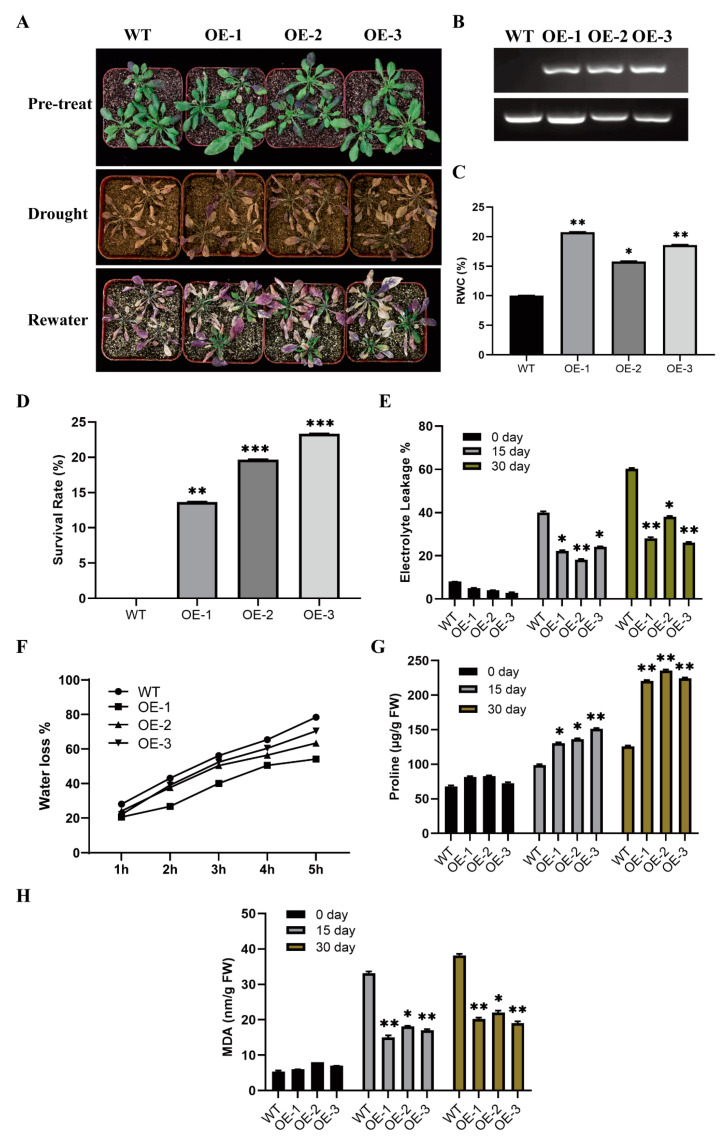
Drought tolerance analysis in GhWOX4 overexpressed transgenic *Arabidopsis* lines. (**A**) Phenotypic comparison of seedlings grown to observe drought tolerance. (**B**) GhWOX4 expression in WT and OE lines analyzed by semi-quantitative (RT) reverse transcription PCR. (**C**) Measurement of relative water content percentage. (**D**) Survival rate percentage after 30 days natural drought and 1 week re-water. (**E**) Electrolyte leakage percentage. (**F**) Water loss percentage. (**G**) Proline content. (**H**) Melondialdehyde (MDA) content. The data represent the means ± SE from three independent experiments, t-tests: * *p* < 0.05, ** *p* < 0.01, *** *p* < 0.001.

To investigate the function of GhWOX4 in plants, GhWOX4 was introduced to *Arabidopsis thaliana* (col-0). Twelve independent overexpressed (OE) transgenic-lines of GhWOX4 were generated in *Arabidopsis*. The semi-quantitative reverse transcription polymerase chain reaction (RT-PCR) verified that GhWOX4 was well expressed in three independent transgenic lines, OE1, OE2, and OE3, which were selected for succeeding experiments. No amplification was observed in wild type control (columbia-0) plants (Figure 3B). Three weeks old plants were subjected to natural drought treatment for 30 days then re-hydrated for 1 week to examine drought response and survival rate (Figure 3D). The transgenic plants responded well, and re-growing was started when they were re-watered (Figure 3A). Relative water content (RWC) percentage was higher, but water loss rate was much lower in OE lines compared to control plants (Figure 3C,F), indicating transgenic lines were more likely to retain water when subjected to drought stress. Electrolyte leakage (EL%) was much higher in control plants as compared to OE lines (Figure 3E). Proline concentration turned out to be significantly higher in OE lines, which indicated higher oxidative and osmotic stress protection in transgenic lines as compared to control (Figure 3G). Furthermore, significantly higher melondialdehyde (MDA) concentration in control lines indicated that more cell damage occurred due to drought stress in control (wild type) plants as compared to OE lines (Figure 3H). Resultantly, overexpression of GhWOX4 in *Arabidopsis* increased tolerance to drought stress.

### 2.4. Knock-Down of GhWOX4 Bottled-Up Tolerance to Drought Stress

**Figure 4:**
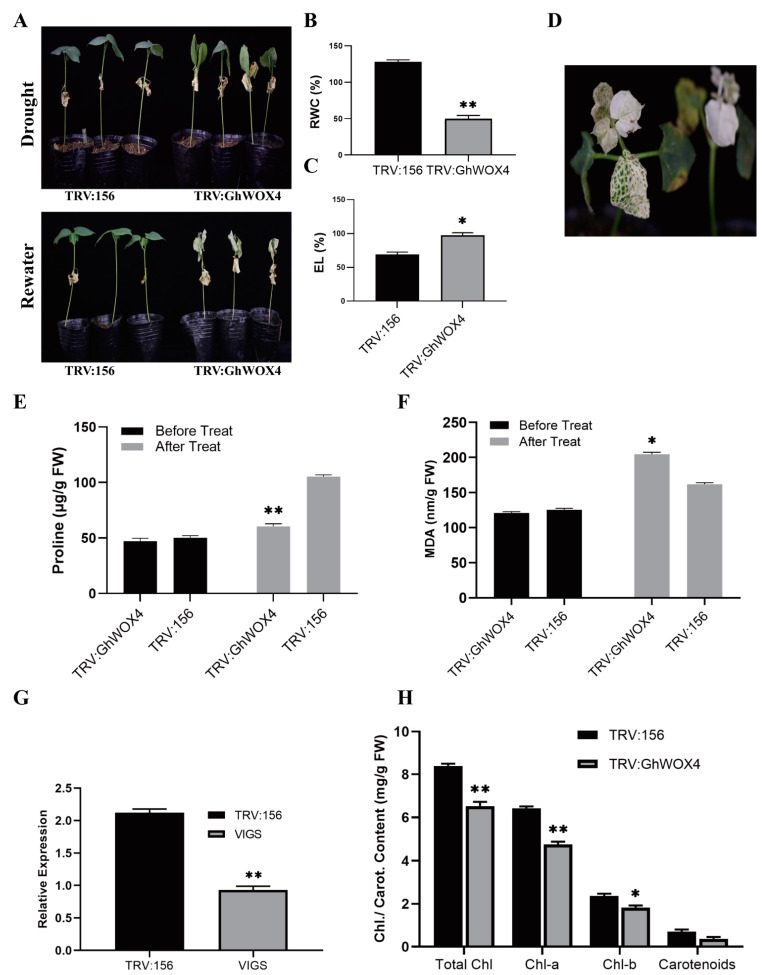
Silencing of GhWOX4 compromised cotton drought stress tolerance. (**A**) Soil grown virus-induced gene silencing (VIGS)-cotton plants subjected to natural drought treatment. (**B**) Measurement of relative water content percentage. (**C**) Electrolyte leakage percentage measurement. (**D**) Albino marker plant. (**E**) Proline content. (**F**) MDA content. (**G**) Relative silencing efficiency, VIGS, and control plants analyzed by q-RT PCR. VIGS expression was declined to less than half of the control plants (**H**) Chlorophyll content and carotenoids content. The data represent the means ± SE from three independent experiments, t-tests: * *p* < 0.05, ** *p* < 0.01.

The role of GhWOX4 in response to drought stress in cotton was investigated. Virus induced gene silencing (VIGS) technology was utilized to decline the endogenous gene expression in cotton plants. The emergence of albino phenotype on pYL156-PDS cotton plants was regarded as a success of the experiment (Figure 4D). The qRT-PCR was used to determine the expression levels of GhWOX4 in VIGS (pYL156-GhWOX4) and empty vector (pYL156) control plants. Considerably reduced expression of the GhWOX4 gene (Figure 4G) in the VIGS plants as compared to the control plants validated successful knocked-down of the gene. The empty control and VIGS plants were exposed to natural-drought stress for 1 week; the VIGS plants appeared with critical wilting phenotype on leaves compared to that of the empty control plants, as shown in Figure 4A. Moreover, these cotton plants were examined for water holding assays. VIGS plants could not managed to retain water content and started wilting earlier than control plants (Figure 4A,B). Similarly, electrolyte leakage percentage was higher in VIGS plants as compared to control plants (Figure 4C). Furthermore, to investigate the obscure potential mechanisms negatively affecting the desiccation tolerance in VIGS plants, MDA and proline contents were analyzed. Under the condition of normal growth, no difference was observed, whereas a spiked accretion of MDA and proline content was observed under water deficient conditions. The VIGS plants accumulated more MDA content than that of the pYL156 control plants, indicating there was more cell damage due to drought stress that caused poor drought tolerance in silenced plants (Figure 4F), whereas less proline content was detected in the VIGS plants than that of the pYL156 control, indicating there was less cell protection and ROS scavenging activity in the VIGS plants as compared to the control (Figure 4E). Furthermore, total chlorophyll content along with carotenoid content were measured. Significantly lower concentrations of these photoreceptors revealed that less light harvesting activity and resultantly less photosynthetic activity occurred in VIGS plants (Figure 4H). Both these indicators were significantly higher in pYL156 control as compared to VIGS plants, which also implies that light may have an interlinked effect with those of plant growth and drought stress.

### 2.5. Knock-Down of GhWOX4 Inhibits the Vascular Cell Growth

**Figure 5:**
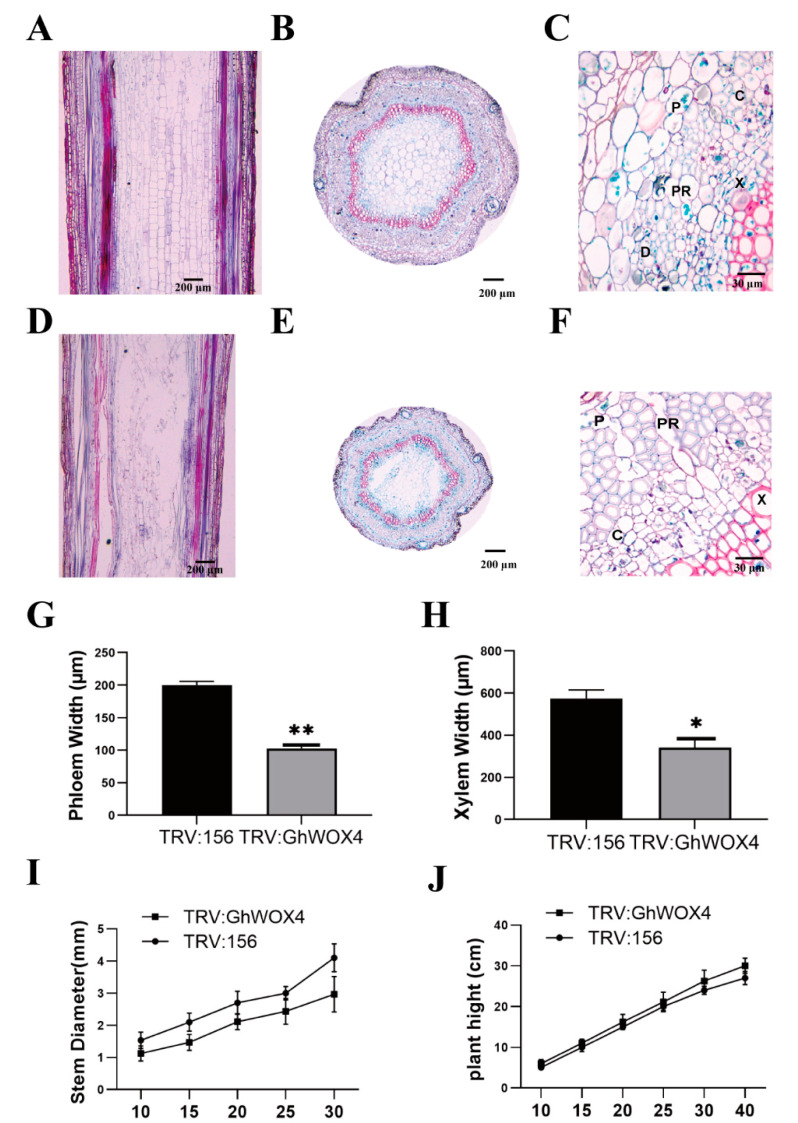
Reduced vascular region and cambial division in TRV:156 (control) and TRV:GhWOX4 (VIGS). (**A**) Stem-control (CK) longitudinal cross-section. (**B**) Stem (CK) transverse cross-section. (**C**) Anatomical close up of CK plant. (**D**) Stem (VIGS) longitudinal cross-section. (**E**) Stem (VIGS) transverse cross-section. (**F**) Anatomical close up of VIGS plant. (**G**) Phloem width in the stem of CK and VIGS plant. (**H**) Xylem width in the stem of CK and VIGS plant. (**I**) Stem diameter comparison of CK and VIGS plant. (**J**) Plant height comparison of CK and VIGS plant. Bar = 200 µm or 30 µm. C, cambium; P, phloem; X, xylem; PR, phloem ray; D, division activity. The data represent the means ± SE from three independent experiments, t-tests: * *p* < 0.05, ** *p* < 0.01.

Drought responsive results in OE transgenic lines and knocked down plants encouraged us to perform anatomical study. A significant difference between stem diameter of TRV: 156 (control) and GhWOX4-silenced plants was detected (Figure 5I), but no significant difference in plant height (Figure 5J) was observed. Microscopy technique was used to observe different cross-sections of control and silenced plants. In TRV: GhWOX4 plants, cambium width was reduced, with less dividing activity and poor development than that of control plants (Figure 5C,F). Furthermore, xylem and phloem area was petite, and cells were looking shrunk in TRV:156 (control) as compared to TRV: VIGS plants (Figure 5G,H). Our results were in line with previously reported studies [11,13,14,15]. Longitudinal cross section of plant stems showed that arrangement of cell layers and cell area was significantly disturbed in GhWOX4-VIGS plants in contrast to control plants (Figure 5A,D). The crucial role of GhWOX4 in vascular cell proliferation and cell division was also observed in cotton plants. The clear distortion in cell arrangements reconfirmed the genetic requirement of GhWOX4 in vascular growth and development (Figure 5B,E). The negatively affected vascular area in VIGS-cotton plants also directed towards the adverse effect on vascular communication as well as vascular transportation system. This may have compromised drought tolerance in VIGS-cotton plants as compared to the control plants. To evaluate this regulatory mechanism, we performed a comparative transcriptome analysis.

### 2.6. Transcriptome Analysis and Result Summary

To further investigate the regulatory mechanisms of GhWOX4, transcriptome analysis was adopted for TRV: 156 (control) and TRV: GhWOX4 (VIGS) under normal (0 h) and drought stress conditions (1 h). GhWOX4 was a quick-responsive gene and appeared with high expression at 1 h (Supplementary Figure S1). The samples were taken at specified time points to perform the transcriptome analysis.

Details of all the pooled RNA-seq read counts are given here. Data were sampled and analyzed with three replications to enhance the reliability of data. Map rate percentage of TRV: 156-0 h was more than 95% in three replications, and map rate percentage of TRV: 156-1 h in three replications was above 94%. Similarly, map rate percentage of TRV: GhWOX4-0 h was higher than 95%, and map rate percentage of TRV: GhWOX4-1 h was also more than 95% in all three replications. The GC percentage (amount of guanine and cytosine) for all the samples was above 45 (Table 1). This transcriptome data were deemed worthy for succeeding analyses.

### 2.7. mRNA-Seq Revealed Differentially Expressed Genes (DEGs)

**Figure 6:**
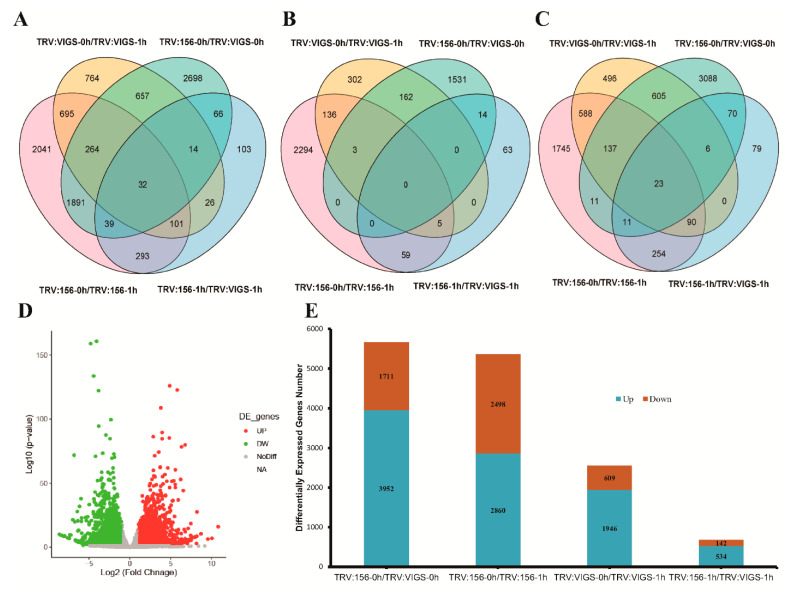
Details of RNA-seq data differentially expressed genes (DEGs) in four comparison sets. (**A**) Venn diagram showing overlapping of total DEGs among four comparison sets. (**B**) Overlapping of downregulated genes. (**C**) Overlapping of upregulated genes. (**D**) Volcano plot, identification of DEGs by Log2FC and q-value (FDR) ≤ 0.05. (**E**) Bar graph representing four combinations; total, blue color representing up, Red-accent2 color denoting downregulated DEGs in each combination group.

Transcriptome-seq data were used for this study to clearly differentiate the over-induced and the under-induced DEGs. Volcano plot analysis was done to identify the changes in sequencing data. Green dots indicated downregulated genes, and red dots represented upregulated genes, whereas genes that were not differentially expressed were denoted with grey dots (Figure 6D). The mRNA-seq data were analyzed in four combination sets: TRV: 156-0 h/TRV: VIGS-0 h expressed a total of 5662 (3952 upregulated and 1711 downregulated) DEGs. However, TRV: 156-0 h/TRV: 156-1 h expressed a total of 5357 (2860 up, 2498 down) DEGs. Likewise, TRV: VIGS-0 h/TRV: VIGS-1 h were set to express a total of 2554 DEGs, of which 1946 were upregulated and 609 DEGs were downregulated. Moreover, TRV: 156-1 h/TRV: VIGS-1 h expressed 675 DEGs in total, representing 534 upregulated and 142 downregulated DEGs (Figure 6E). A Venn diagram of total differentially expressed genes explained 695 DEGs were overlapped between TRV: VIGS-0 h/TRV: VIGS-1 h in comparison to TRV: 156-0 h/TRV: 156-1 h, where 2041 DEGs were unique in TRV: 156-0 h/TRV: 156-1 h. The genes DREB3, DREB1D, and E3 ubiquitin-protein ligase UPL2 were among them (Figure 6A). In upregulated comparison sets, 588 genes were overlapped between TRV: VIGS-0 h/TRV: VIGS-1 h and TRV: 156-0 h/TRV: 156-1 h, where 1745 DEGs were unique in TRV: 156-0 h/TRV: 156-1 h. The genes POX2 and HASPIN were among them (Figure 6C). The downregulated comparison sets expressed that 136 DEGs were overlapped between TRV: VIGS-0 h/TRV: VIGS-1 h and TRV: 156-0 h/TRV: 156-1 h; *thiG*, *MIMI_L27,* and *DUF* were among them (Figure 6B). DEGs details are given in Supplementary File S1.

### 2.8. GO Analysis Revealed GhWOX4 Biological Functions

**Figure 7:**
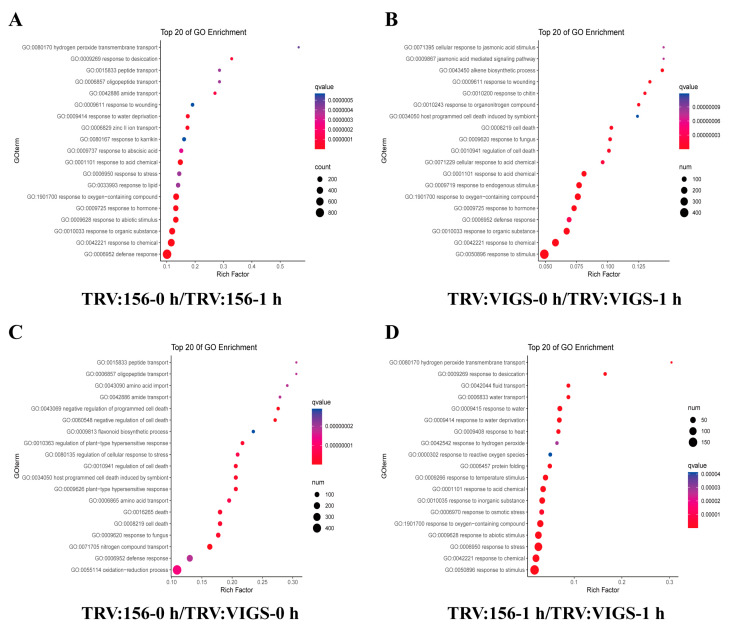
Gene ontology (GO) enrichment analysis. (**A**) GO terms of TRV:156-0 h/TRV:156-1 h (**B**) GO terms of TRV:VIGS-0 h/TRV:VIGS-1 h (**C**) GO terms of TRV:156-0 h/TRV:VIGS-0 h (**D**) GO terms of TRV:156-1 h/TRV:VIGS-1 h. Each circle in the figure represents a GO biological process, and the number of genes enriched in a pathway corresponds to the size of the circle. The degree of significance of the enrichment of DEGs in a pathway is represented by qvalue. The abscissa indicates the ratio of the number of DEGs annotated to a particular GO term to the number of the DEGs annotated to all GO terms.

To get into the details of categories represented by differentially expressed genes, the GO enrichment analysis was performed. The GO terms corroborated a number of biological processes (BP) uniquely over-represented in TRV: 156-0 h/TRV: 156-1 h in drought stress condition. Different terms related to defense response, secondary metabolites, and oxidation reduction were enriched in both VIGS as well as in control, which indicated they were common processes. However, the GO processes GO:0009269 response to desiccation, GO:0009414 response to water deprivation, GO:0008283 cell proliferation, GO:0051726 regulation of cell cycle, GO:0009725 response to hormone, GO:0009737 response to abscisic acid, GO:0006829 zinc II ion transport, GO:0015833 peptide transport, and GO:0006950 response to stress were significantly and uniquely enriched in the genes of TRV: 156-0 h/TRV: 156-1 h with elevated expressions as compared to TRV: VIGS-0 h/TRV: VIGS-1 h (supplementary File S2). The top 20 enrichment terms are shown in Figure 7. Similarly, GO terms associated with photosynthesis, light response, metabolism, and transport were highly enriched in the TRV: 156-0 h/TRV: 156-1 h (Figure 7A). GO terms GO:0015833 peptide transport, GO:0006857 oligopeptide transport, GO:0043090 amino acid import, GO:0042886: amide transport, GO:0006865 amino acid transport, GO:0071705 nitrogen compound transport, GO:0006952 defense response, and GO:0055114 oxidation-reduction process were also enriched in the DEGs of TRV: 156-0 h/TRV: VIGS-0 h, suggesting that potential of transport and defense response was changed when GhWOX4 was silenced without drought stress (Figure 7C). GO terms GO:0009269 response to desiccation, GO:0006833 water transport, GO:0009414 response to water deprivation, GO:0006970 response to osmotic stress, GO:0009628 response to abiotic stimulus, and GO:0006950 response to stress were significantly enriched in TRV: 156-1 h/TRV: VIGS-1 h, suggesting that potential of water transport and stress response was changed when GhWOX4 was silenced along with drought stress (Figure 7D). A wide range of these functional annotations were evident for the imperative role of GhWOX4 in drought stress alongside vascular cells growth and development. These GO terms were absent or under-induced in TRV: VIGS-0 h/TRV: VIGS-1 h (Figure 7B) as compared to the TRV: 156-0 h/TRV: 156-1 h. Our results suggest that a generous number of DEGs involved in transport and signaling response may be regulated by GhWOX4 to form a complex plant-growth-stress-tolerant regulatory network.

### 2.9. TFs and Hormones Play Key Roles in GhWOX4 Mediated Plant Growth and Drought Tolerance

**Figure 8:**
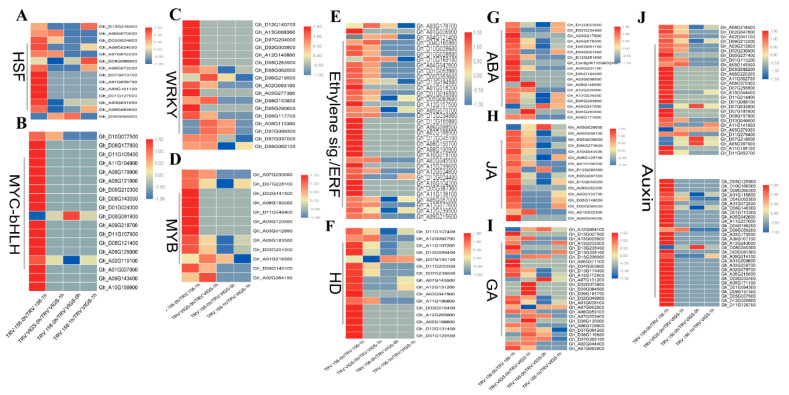
Heat maps indicating expression comparison of hormones or transcription-factors among four comparison sets. (**A**) Heat shock transcription factor (HSF). (**B**) MYC-bHLH. (**C**) WRKY. (**D**) MYB. (**E**) Ethylene signaling and response-factors. (**F**) Homeobox domain transcription factors (TFs). (**G**) Abscisic acid (ABA). (**H**) Jasmonic acid (JA). (**I**) Gibberellic acid (GA). (**J**) Auxin. TBtools-software was used to construct heatmaps, Red color represents upregulation, and blue color represents downregulation.

The enriched GO terms revealed that an extensive number of differentially expressed genes related to TFs and phytohormones were uniquely and highly induced in the TRV: 156-0 h/TRV: 156-1 h in contrast to the TRV: VIGS-0 h/TRV: VIGS-1 h under drought stress (Supplementary File S1). Additionally, part of these DEGs included those related to biosynthesis, metabolism, action of auxin (IAA), abscisic acid (ABA), ethylene, and gibberellin (GA) (Figure 8E,G–J). Auxin was one of the over-represented and differentially expressed hormones. The signal transduction involved 60 DEGs, including the auxin response-factor (ARF) family, the auxin responsive protein (AUX/IAA) family, the amino acid transporter protein (AUX1) family, the GH3 auxinresponsive promoter (GH3) family, and the auxin responsive SAUR protein (SAUR) family. For the ABA signal transduction pathway, six gene families were identified, including the PYL/PYR (abscisic-acid receptor) family, the Serine-threonine protein kinase (SAPK) family, the protein phosphatase 2C (PP2C) family, the CBL-interacting protein kinase (CIPK) family, the calcium-dependent protein kinase (CDPK) family, and the calmodulin (calmodulin-like protein) family. DEGs associated with ethylene signaling, including ERF and AP2-EREBP, were also observed with high expression. Some representative members of these hormones—auxin (AGD1,5, ARF5, ARF7, SAUR72), ABA (ABF3, AIP1, CYP707A1, PYL9), jasmonic acid (JA)/TIFY (TIFY6B, TIFY11B, TIFY10A), ethylene (ERF109, ERF1A, ERF4, ERF053), and GA (GA2OX8, GASA4, GA2OX1)—were amongst the highly enriched DEGs (Supplementary File S1). It suggested that phytohormones have a vital role to play in the regulation of defense and plant growth.

A number of TF-families were induced in exchange against drought stress, and a higher number of transcription factors (TFs) was observed to be induced in TRV: 156-0 h/TRV: 156-1 h as compared to the TRV: VIGS-0 h/TRV: VIGS-1 h under drought stress. These families include: MYB, AP2-ERF, MYC-bHLH, HB, bZIP, WRKY, HSF, GRAS, NAC, LOB, AUX/IAA, and C2C2-Dof (Figure 8A–F). Some members of the over-represented TFs involved, such as MYB (88, 44, 2, 17, 105), WRKY (70, 2, 7), MYC (MYC2, MYC4, LHW, b-HLH13, b-HLH14), and homeodomain (ATHB7, ATHB8, WOX3, WOX4, ANL2) were differentially or uniquely induced in the TRV: 156-0 h/TRV: 156-1 h as compared to the TRV: VIGS-0 h/TRV: VIGS-1 h. These results indicated that GhWOX4 modulates an ample number of TFs and hormones to respond to drought stress as well as plant growth and development related pathways. Silencing of GhWOX4 adversely affected related transcription and signaling pathways, which highly compromised the plant drought stress response as well as plant growth in return (Figure 8) (Supplementary File S1). This suggested that many TF genes contribute in early gene (GhWOX4) activation in cotton.

### 2.10. Insilico Promotor Analysis Identified the Important Cis-Acting Regulatory Elements (CAREs)

**Figure 9:**
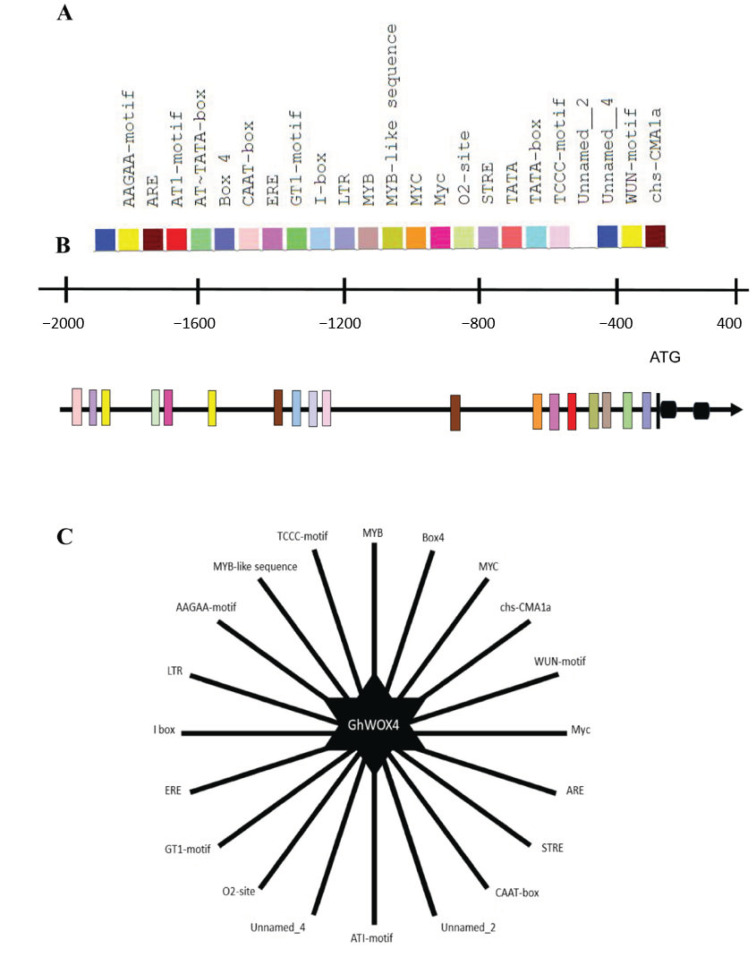
In silico promotor analysis. (**A**) Plantcare-database derived cis-regulatory elements (CREs). (**B**) Position of CREs, 2000 bp upstream to ATG (start codon) with respect to scale; black-filled terminator represents “ATG”, black rectangles represent exons, black horizontal lines represent introns. (**C**) Illustration representing total CREs reported in the promotor region of GhWOX4.

The expression of important TFs, signaling hormones, and light responsive genes prompted us to explore the promotor region of GhWOX4. To understand the transcriptional regulations, in silico promotor analysis was executed. Several potentially inducible cis-regulatory elements (CREs) were reported. MYB, MYB-like, myc, and MYC recognition sites were reported to function as cis-acting regulatory elements (CAREs) in the dehydration-tempted expression of the drought responsive genes [21]. Likewise, CREs for anaerobic induction and low temperature were also reported. CREs and their respective positions are given in (Figure 9). ARE (auxin responsive) and ERE (ethylene responsive) motifs underlying different stress and growth signaling pathways were also present. Similarly, light-response related cis-regulatory elements were abundantly present, such as GT1, I-box, and box-4, required for the transcriptional regulation of light-responsive genes. Functional details and abundance of all CREs are given in Supplementary Table S1. Less representation of these genes was observed in TRV: VIGS-0 h/TRV: VIGS-1 h as compared to TRV: 156-0 h/TRV: 156-1 h.

The irreplaceable role of light in plant growth and development is well reported. We surveyed RNA-seq data and discovered light responsive DEGs. A class of photoreceptors in plants, phytochromes (Phys), gets activated by light and senses light and temperature. Cytochromes (CYPs), which are involved in electron transport chain and play an integral role in photosystem (PS), were differentially expressed in TRV: 156-0 h/TRV: 156-1 h as compared to TRV: VIGS-0 h/TRV: VIGS-1 h (Supplementary Figure S2). Similarly, a higher number of GO terms was enriched related to transport and energy. It indicated that DEGs related to heat and light responsiveness were over-induced in the TRV: 156-0 h/TRV: 156-1 h in comparison to the TRV: VIGS-0 h/TRV: VIGS-1 h, e.g., heat-shock transcription factors (HSFs), heat-shock proteins (HSPs) (Figure 8), photoreceptors, Phys, and CYPs (Figure S2). Silencing of GhWOX4 might have interrupted the light harvesting, which headed towards lack of required energy for vascular signaling and material flow.

## 3. Discussion

Cotton growth and yield are extremely affected by drought. To tackle this problem, we need better understanding of drought responsive pathways. To investigate this, we selected a quick-responsive and highly expressive TF under water scarcity, GhWOX4. GhWOX4-overexpressed transgenic and VIGS lines were constructed. Drought stress assays proved that GhWOX4 positively regulated drought tolerance. We hypothesized that GhWOX4 may mediate drought tolerance through plant vasculature because the plant stem girth of GhWOX4-silenced lines was significantly reduced from that of control plants. Tissue microscopy of stem cross-sections (GhWOX4-silenced) revealed the prime role of GhWOX4 in vascular cell proliferation and growth. In accordance to previous studies [14,15], silenced plants developed significantly petite vascular areas. The phloem, xylem, and cambium zone was shortened, with squeezed cells and lesser cell-dividing activity in GhWOX4-silenced plants (Figure 5A–F) in comparison to TRV: 156 (control). To further investigate the regulatory mechanisms, GhWOX4 was coupled to regulate drought stress and vascular growth and development. Comparative transcriptomic data analysis was done that laid out biochemical and metabolic processes observed in both TRV: 156-0 h/TRV-156-1 h and TRV: VIGS-0 h/TRV: VIGS-1 h combinations. Plants use ROS scavenging enzymes to counter the oxidative damage in drought stress [22], but enhanced ROS-scavenging activity was observed in TRV: 156-0 h/TRV: 156-1 h in contrast to the silenced set, which was also proved in Figure 3 and Figure 4 assays. Related GO results are given in Supplementary File S2. Silencing of GhWOX4 tempted the expression of CBL-interacting serine/threonine-protein kinase 6 (CIPK), which involved water deprivation response, ATP binding, and auxin transport [23] un-induced in TRV: VIGS-0 h/TRV: VIGS-1 h (silenced) plants in contrast to control.

Under the molecular basis for drought tolerance, considerable amounts of DEGs were induced after drought treatment. The GO terms analysis showed that numerous DEGs related to inter/intra cell transport, light response, response to lipid, flavonoid biosynthesis, transcription, signaling, etc., were significantly enriched in DEGs of TRV: 156-0 h/TRV: 156-1 h as compared to TRV: VIG-0 h/TRV: VIGS-1 h, suggesting that stress response and plant growth potential were changed due to GhWOX4 loss of function. This is consistent with previous studies, where proteins related to these biological processes were induced in phloem in response to drought stress [24,25,26]. Pointedly, some previously reported important hormones as well as TFs homologues were also found in our study.

Transcription factors play a chief role in abiotic stress responses as well as in the regulation of various biological processes, e.g., MYB88, which is involved in water deprivation, cell differentiation, and root development [27]. MYB44 is involved in drought stress and hormonal signaling pathway. MYB16 is involved in cell morphogenesis and trichrome development. Similarly, MYB61, MYB102, MYB103, MYB17, MYB16, MYB30, MYB105, etc., [28,29] were among the uniquely expressed genes in TRV: 156-0 h/TRV: 156-1 h as compared to TRV: VIGS-0 h/TRV: VIGS-1 h.

BZIP10, a member of basic leucine zipper TF, is involved in plant immunity and abiotic stress responses [30]. BZIP44 helps in DNA binding and root growth [31]. RF2b is a transcription factor involved in vascular development and shoot-tissue organization [32]. ABF2 is involved in ABA-regulated stress responses and turned out to be a positive constituent of glucose signal-transduction [33]. Transcription factors MYC2, MYC4, and MYC3 are involved in desiccation response ATP and hormone signaling [34]. RVE7 is involved in circadian rhythm and hormone response [35]. KUA1 is involved in chloroplast function and responses to light and sugar and promotes responses to auxin, abscisic acid (ABA), and ethylene [36]. A versatile TF, WRKY70 is involved in hypoxia, water deprivation, ethylene, and JA in conjunction with WRKY54 and WRKY46 and stimulates brassinosteroid-regulated plant growth by modulating gene expression [37]. WRKY2 regulates cell division and other TFs [38]. WRKY23, WRKY6, WRKY7, etc., [39] were uniquely induced in TRV: 156-0 h/TRV: 156-1 h. Other TFs such as, ATHB7, ATHB8, HAT3, HAT22, WOX3,4, and ANL2 [40] were among some TFs described here (Figure 8A–E) that were uniquely and differentially expressed in TRV: 156-0 h/TRV: 156-1 h as compared to TRV: VIGS-0 h/TRV: VIGS-1 h. This suggests that these TFs may collaborate with GhWOX4 to regulate plant growth and drought tolerance.

Phytohormones are involved in plant response to drought stress, as they help to regulate plant growth and drought stress [41]. Auxin signaling is mediated by ARFs (auxin response factors), and auxin fosters the expression of HD (homeodomain) TFs, particularly ATHB8 and WOX4, to regulate the vascular tissues development [20]. Likewise, ABA is involved in hormone homeostasis with auxin and contributes in drought response and vegetative growth and regulates sugar and macromolecule transport in vascular tissues [42]. Studies reported that ethylene contributes in drought stress response, light signaling, and plant growth processes. ERF contributes in the plant cell division pathway with WOX4/14 [43,44]. GhWOX4 may amalgamate with these hormones to fine-tune plant growth and desiccation response [6].

As reported in one study, “the Xylem and the phloem pathways develop a signaling feedback circuit in plants, signal molecules are transmitted across long-distances to response the environment via vascular tissues” [10]. With the help of turgor pressure and energy, sugars and peptides are transported to other plant organs; xylem moves fluids and signals from the roots, while phloem intercedes the auxin transport and is highly important in sculpting the plant vascular tissues development [45,46]. Knock-down of GhWOX4 interrupted the development of vascular tissues in GhWOX4-silenced plants as compared to control plants [11,15]. Thus, transport potential was inhibited, which caused the drought sensitive phenotype [8,10].

During photosynthesis, chloroplasts convert the light energy into chemical energy and tend to use this for plant growth and development [47]. Drought stress causes production of O2 (ROS) in the chloroplast that can capture the electrons from the photosynthetic electron transport chain (PETC) [42], which leads to oxidative damage in the plasma membrane and photosynthetic pigments [48]. A maintained photosynthetic rate in water scarcity condition not only pertains to photosynthetic capacity but helps in drought tolerance as well [41,49]. GhWOX4 loss of function reduced the chlorophyll and the carotenoid contents (Figure 4H), which led to low light harvesting and photosynthesis rates and, consequently, less drought tolerance and plant growth.

Gene expression is largely regulated at the transcriptional level by gene promotors [21]. Putative receptor sites in the GhWOX4 promotor provide intimations about the potential contributors involved in the under-study regulatory network. Several well known binding elements related to drought response, light response, and plant growth were found, such as Wun-motif, STRE, I-box, MYC, MYB, ARE, ERE, etc. [34,47,50]. Presence of imperative cis-acting regulatory elements (Supplementary Table S1) in the upstream region of GhWOX4 indicated it liaises stress response and plant growth via hormonal signaling and transcriptional activities. However, silencing of GhWOX4 may interrupt the expression and the pathways featuring these cis-regulatory elements [47].

**Figure 10:**
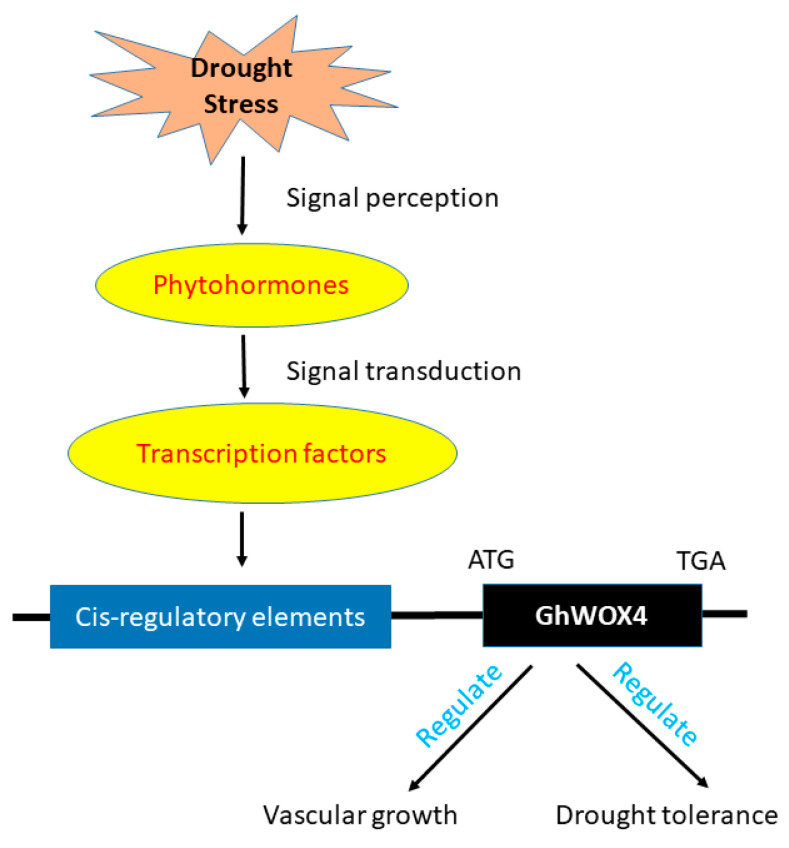
Model for GhWOX4 function in cotton, regulating drought stress tolerance and vascular growth by collaborating with different CREs (cis-regulatory elements), phytohormones (auxin, ethylene, ABA, etc.) and transcription factors (MYB, MYC-bHLH, WRKY, HSF, HD, etc.). Expression of GhWOX4 enhances in drought stress by affecting vascular growth to strengthen drought stress tolerance and plants growth maintenance.

Taken together, these results led us to the conclusion that GhWOX4 is a master regulator that amalgamates myriad TFs, phytohormones, and photosystem components to regulate plant growth and stress response (Figure 10). Nonetheless, further studies are needed to elaborate GhWOX4-mediated cross-talks among different pathways.

## 4. Materials and Methods

### 4.1. Plant Material and Growth Conditions

Upland cotton variety “Zhonmiansou 24” was used in this study. To promote germination, seeds were washed and soaked in water, then placed at room temperature (RT) for 2 days in the dark. Uniform seedlings were grown in pots filled with vermiculite and soil (*v*/*v* = 1:1). Then, they were grown under 16 h light/8 h dark photoperiod with 30 °C temperature in the greenhouse. Seedlings were applied with 15% polyethylene glycol 6000 (PEG_6000_) for drought treatment, then leaves were harvested at 0, 1, 3, 6, 12, 24 h and immediately chilled in liqued-N_2_ and stored at −80 °C to extract RNA. Three weeks old *Arabidopsis* plants were exposed to natural drought treatment for 30 days, then re-watered for one week to examine the response. *Arabidopsis thaliana* were grown in vermiculite/nutritious soil = 1:1. To grow on agar plates, sterilized seeds with 50% sodium hypoclorite (NaClO) were vernalized at 4 °C in the dark for 2 days and then placed in an artificial growth chamber under previously explained photoperiod at 20–21 °C temperature.

### 4.2. Cloning and Ectopic Expression of GhWOX4

Full length coding-sequence (cds) was used to clone GhWOX4 with specific primers (Table S1). The PCR product was ligated to the entry vector pQBV3 with EcoRV linkers. The plasmid was transformed into *Escherichia coli* (DH5α strain) competent cells. This construct was recombined into the plant binary vector pEarleyGate101 (containing Basta resistance gene and yellow fluorescent protein) with LR clonase (Invitrogen, Carlsbad, CA, USA). To study overexpression, the 35S::GhWOX4 vector was constructed by digesting the GhWOX4-CDS with Xba I and BAMH I. *Agrobacterium* (strain GV3101) mediated transformation via electroporation was done. Transgenic OE lines were generated using floral-dip method in *Arabidopsis* [51]. Basta-herbiside and cephalosporin contained MS medium (Murashige & skoog) was used to select the positive lines. Seedlings of T_2_ generation were selected following 3:1 segregation ratio. The seedlings of T_3_ generation (homozygous) were reconfirmed by qRT-PCR and used for subsequent experiments.

### 4.3. RNA Extraction and Expression Analysis by qRT-PCR

To isolate total RNA, the RNAprep PurePlant Kit was used as per the manufacturer’s instructions (Tiangen, Beijing, China). The synthesis of cDNA was done using Prime-Scrip^TM^ II1st Strand cDNA-Synthesis Kit (Takara Bio, Dalian, China). For semi-quantitative reverse transcription PCR (RT-PCR), given parameters with DNA polymerase (TKS Gflex) were used, i.e., 94 °C for 5 min, 27 cycles of 15 s at 98 °C, 15 s at 60 °C, and 30 s at 68 °C. Agarose (1%) gel was used to assess the PCR products. The qRT-PCR was executed on the “ABI7900 hT system” (Applied-Biosystems, Foster City, CA, USA) with the help of SYBR Premix Ex-Taq Kit. A 3-step method was practiced with the following PCR conditions: 40 cycles at 95 °C for 30 s, 95 °C for 5 s, and 60 °C for 30 s. For each reaction, the dissociation curve was checked, and CT-method (2^−∆∆Ct^) was used to quantify the expression level of the target gene [52]. For internal control, Gh-Actin and At-Actin genes were used. Primers used in present study are listed in (Supplementary Table S2).

### 4.4. TRV-Mediated Gene Silencing and Drought Assay

A 654 base pairs fragment of GhWOX4 was amplified and implanted into the XbaI and the BamHI restriction sites of the pYL156 vector. The recombinant vectors were transformed into *Agrobacterium* (GV3101 strain). The GV3101 carrying vector components pYL156 (empty vector), pYL156-CLA1 (positive control), pYL156-GhWOX4 (VIGS), and pYL192 (helper vector) were shaken in Luria Bertani (LB) medium containing 50 µg/mL rifampicin, gentamycin, and kanamycin by means of a shaker at 28 °C with 180 RPM. The culture was monitored when the OD_600_ value of the culture reached 1.5–2.0. Then, the culture was taken off from the shaker, centrifuged, then re-suspended, and the OD_600_ value was adjusted to 1.8 with infiltration buffer (10 mM MES, 200 µM acetosyringone and 10 mM MgCl_2_) solution. The suspension was left in the dark for 3 h at room temperature. Before injection, the suspensions of pYL156 (EV), pYL156-GhWOX4 (VIGS), and pYL156GrCL1 (PDS) were mixed with pYL192 in a 1:1 ratio. For each cotyledon, the underside of the leaf was injected with mixture solution. The injected seedlings were left in the dark overnight and transferred to a greenhouse at 25 °C with a 12 h light/12 h dark cycle. The plants were injected with pYL156 (empty control) and pYL156-GhWOX4 and were exposed to drought treatment after 4 weeks. Two methods were used to apply drought treatments. The pYL156 (EV) and the pYL156-GhWOX4 (VIGS) plants were irrigated with 15% (*w*/*v*) PEG_6000_ or were subjected to natural drought treatment to examine their drought response. Every experiment was performed with at least three biological replications.

### 4.5. GhWOX4 Subcellular Localization

Amino acid sequence analysis of GhWOX4 picked out the nuclear signal peptide. To verify this nuclear localization, first of all, the gene (QBV3GhWOX4) was cloned, ligated into the QBV3 vector, and lastly constructed into the pEG101 (YFP fluorescent tag-containing) vector to generate expression vector GhWOX4-YFP [19]. The tobacco (*Nicotiana benthamiana*) leaves were injected with *Agrobacterium tumefacians* containing the GhWOX4-YFP vector to identify transient expression. To detect the localization signals 48 h later, the infiltrated *N. benthamiana* leaves were observed via “laser scanning confocal microscope” (OLYMPUS FV1200 confocal microscope). Nuclear localization of GhWOX4 was verified by this method. Genes sequences used in study were obtained from the Cotton-database (https://cottonfgd.org) or https://www.cottongen.org [2].

### 4.6. Physiological Indices

For relative water content (RWC), 15 cotton leaves from each group were weighed (FW) after harvesting from the plant and put into distilled water for 4 h and then turgid weight (TW). The leaves were desiccated in an oven at 80 °C for 24 h to take their dry weight (DW). The given formula was utilized to calculate the RWC% = (FW − DW)/(TW − DW) × 100. The water loss rate was measured by applying the leaves with desiccation for 5 h. The plants were subjected to drought either by withholding of water (natural drought) or by applying PEG_6000_ (*w*/*v*) [53]. Using 0.1 g of biomass (leaf tissues) malondialdehyde (MDA) and proline were quantified with the help of “assay-kit” (Nanjing Jiancheng Bioengineering institute, Nanjing, China) following the prescribed procedure. Electrolyte leakage percentage was measured as described in a previous study [54].

### 4.7. Chlorophyll and Carotenoid Contents

Leaves were used to extract the pigments using 80% (vol/vol) acetone. “The absorbance of acetone extract without cell-debris was measured at wavelength of 480 nm for total carotenoids. The content of total carotenoids was calculated according to [55].

Total Carotenoids (µg mL^−1^) = 4.0 × Abs480 nm,

where Abs480nm is the absorbance of 80% acetone extract measured at 480 nm.

Chlorophyll a, b and total Chlorophyll were evaluated by measuring the absorbance of the acetone extract at 664 nm and 647 nm and calculated according to [56].

Chl a (µg mL^−1^) = (12.25 × Abs664 nm) − (2.55 × Abs647 nm);

Chl b (µg mL^−1^) = (20.31 × Abs647 nm) − (4.91 × Abs664 nm),

Total Chl (µg mL^−1^) = Chl a (µg mL^−1^) + Chl b (µg mL^−1^),

where Abs647 nm and Abs664 nm refer to the absorbance of the 80% acetone extract measured at 664 nm and 647 nm respectively”.

### 4.8. Anatomical Analysis

For anatomical studies, 4 week old cotton plants (control and silenced) were subjected to natural drought treatment for one week, and stem samples were taken. Controlled condition grown cotton stems (5 weeks old) were used to take samples and measurements. The stem diameter of plants was measured at the base of the stem internode 1 cm above the soil surface using a Vernier caliper. To analyze width of tissue layers, 1 cm stem samples were cut and fixed for 24 h in FAA (Formalin-Aceto-Alcohol) solution containing 5% formaldehyde, 5% acetic acid, and 50% ethanol. Then, samples were sequentially desiccated, gradually infiltrated, and embedded using the [13] method. Measurement of xylem and phloem widths was done using Image J software [57,58].

### 4.9. RNA-Seq Library Construction and Data Analysis

For mRNA-Seq analysis, the 4 week old cotton plants (control and silenced) were applied with 15% polyethylene glycol 6000 solution (PEG_6000_) (*w*/*v*). Samples were taken at 0 h as control (4 week old control and GhWOX4-silenced cotton plants without PEG treatment) and 1 h as treated (1 h after PEG treatment). The third true leaves of TRV::00 and TRV::GhWOX4 plants were harvested and directly chilled in liquid-N_2_. The extraction of total RNA was done as previously described.

The binary read alignment files were used as input to Cufflinks [59], which assembled the reads into transfrags (transcripts). The estimated gene abundance was then measured in terms of the (FPKM) fragments per kilobase of transcript per million mapped reads. DEGs between 2 sample sets were identified by Cuffdiff. The lists of significant up/down regulated genes for drought samples were then obtained. Only the genes with a log2 fold change (log2FC) ≥+1 and ≤−1 but without infinite values and FDR adjusted *p* ≤ 0.05 were considered as significantly DEGs. The GO terms with q-value of ≤0.05 were regarded as significantly enriched.

### 4.10. Insilico Promotor Analysis

The promotor sequence (2 kbp) of GhWOX4 was downloaded from cottonfgd.org and was submitted to plantcare cis-acting-regulatory element database (http://bioinformatics.psb.ugent.be/webtools/plantcare/html/) database to identify CAREs in the potential promotor region of the gene [60].

## 5. Conclusions

The results of our study demonstrated that GhWOX4 shares high correspondence with the WOX4 gene in other plant species in terms of protein sequence, sub-cellular localization, and phylogenetic relationship. The critical role of GhWOX4 was established, where overexpressed transgenic lines enhanced drought tolerance. Alternatively, GhWOX4 silencing resulted in enhanced susceptibility to drought stress in cotton plants. A vital role of GhWOX4 in the vascular cell niche was ascertained, as knock-down of GhWOX4 highly relinquished vascular growth and development in plants. RNA-seq approach enhanced our understanding that DEGs related to abiotic stress response, light response, transport, as well as plant growth and development were downregulated in GhWOX4 silenced plants in relation to control. Important hormones and TFs were highly enriched in control plants as compared to GhWOX4 silenced plants. The upstream promotor region of GhWOX4 was found enriched with well known cis-acting regulatory elements related to drought response, light response, plant growth, and development, indicating GhWOX4 integrates multiple pathways to fine-tune drought tolerance and plant growth and development. The present study not only provides insights into the role of GhWOX4 in drought tolerance, plant growth, and development but may be of significance in uncovering the myriad molecular mechanisms GhWOX4 is involved in. This understanding will help in developing and breeding cotton genotypes that can perform well in adverse environments.

## Figures and Tables

**Table 1:** Summary of 12 separately pooled RNA-sequencing read counts, using *Gossypium hirsutum* as a reference genome.

Sample ID	Read Sum	Mapped Read Sum	Map Rate (%)	GC (%)
* PYL_VIGS-0 h_R1	46,162,896	44,371,337	96.12	45.59
PYL_VIGS-0 h_R2	43,242,842	41,587,275	96.17	45.22
PYL_VIGS-0 h_R3	42,443,503	40,570,587	95.59	45.35
PYL_VIGS-1 h_R1	56,984,332	54,501,470	95.64	45.14
PYL_VIGS-1 h_R2	47,557,755	45,492,367	95.66	45.11
PYL_VIGS-1 h_R3	44,546,148	42,544,884	95.51	45.29
* PYL_156-0 h_R1	51,385,206	49,241,433	95.83	45.39
PYL_156-0 h_R2	45,698,425	43,788,845	95.82	45.45
PYL_156-0 h_R3	49,625,416	47,602,859	95.92	45.39
PYL_156-1 h_R1	43,066,450	41,246,640	95.77	45.05
PYL_156-1 h_R2	41,804,713	39,678,862	94.91	45
PYL_156-1 h_R3	47,323,891	45,363,867	95.86	45.32

* PYL_VIGS; TRV:GhWOX4; * PYL_156; Control (TRV:156).

## Data Availability

The data are available in Supplementary Material here.

## Supplementary Materials

Supplementary materials can be found at https://www.mdpi.com/1422-0067/22/2/898/s1. Figure S1: Expression analysis of GhWOX4 under drought stress treatment at different time points. Figure S2: Expression of light responsive DEGs. Table S1: Functional details of cis-regulatory elements found in promotor region. Table S2: List of all the primers used in study. Supplementary File S1: DEGs details used in study. Supplementary File S2: GO terms output.

## Abbreviations

GhWOX	*Gossypium hirsutum* WUSCHEL-Related homeobox
AUX/1AA	Auxin/Indole-acetic-acid
bHLH	Basic-helix-loop-helix protein
WRKY	Transcription factor (TF), partake WRKYGQK heptapeptide at N-terminal
MYB	Myeloblastosis (Avian Myeloblastosis Viral Oncogene Homolog) TF
myc	Melocytomatosis (isolated from an avian myelocytomatosis virus) TF
TRV	Tobacco rattle virus
ERF	Ethylene response factor
YFP	Yellow-fluorescent protein
PDS	Phytoene-desaturase
ROS	Reactive oxygen species
FDR	False discovery rate
PYR/PYL	Pyrabactin resistance1/PYR1; PYR-like (PYL)
CBL	Calcineurin B-like protein
AP2/EREBP	Apetala2/Ethylene response element binding protein
AGD1	ADP-Ribosylation factor GTPase-activating protein domain
ARF	Auxin response factor
ARE	Auxin response element
SAUR	Small-auxin upregulated RNA
ABRE/ABF	ABA-responsive element/ABRE-binding factor
AIP1	Actin-interacting protein1
CYPs	Cytochrome 450 proteins
GA2OX8/GASA4	Gibbrellin 2-Oxidase; Gibbrellic acid stimulated *Arabidopsis*
HB/HD	Homeobox/Homeobox-domain
GRAS	GIBBERELLIC ACID INSENSITIVE (GAI), REPRESSOR OF GA1 (RGA), and SCARECROW (SCR)
LOB	Lateral-organ-boundaries domain
bZIP	Bacic-leucine zipper
ATHB	*Arabidopsis thaliana*-Homeobox
ANL	Anthocyanin-Less
LHW	Lonesome-highway
RF2B	Fertility restorer-B
RVE7	Protein REVEILLE7
HAT	HD-Zip protein
STRE	StuA-response element
MES	Morpholinoethanesulfonic acid
pEG101	pEarleyGate101 (vector)
ATP	Adenosine tri-phosphate
