# Supplementary material for: Transcriptome Analysis Revealed GhWOX4 Intercedes Myriad Regulatory Pathways to Modulate Drought Tolerance and Vascular Growth in Cotton
Source: Int J Mol Sci. 2021 Jan 18;22(2):898. doi: 10.3390/ijms22020898 (PMC7829754; doi:10.3390/ijms22020898)
Supplement: Supplementary file 1 [file ijms-22-00898-s001.zip › Supplementary_ Figures .pdf]

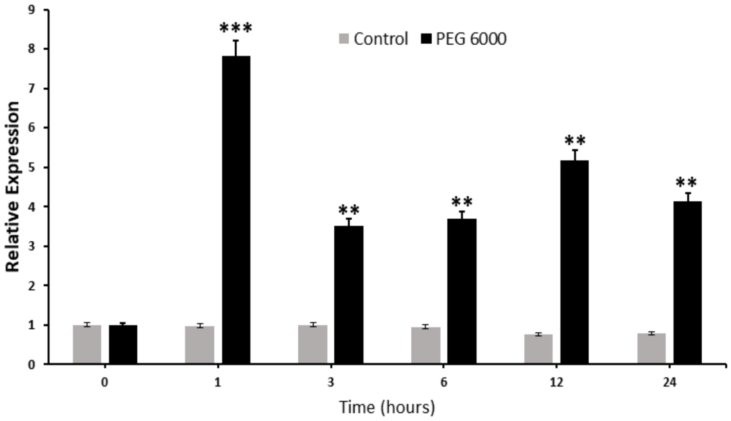


**Supplementary Figure S1. Expression profile of GhWOX4 under drought stress treatment.** Gene expression was determined by qRT-PCR. Relative expression of GhWOX4 under drought treatment at different time points. The data represent the means ± SE from three independent experiments. Independent t-tests: *p < 0.05, **p < 0.01, ***p < 0.001

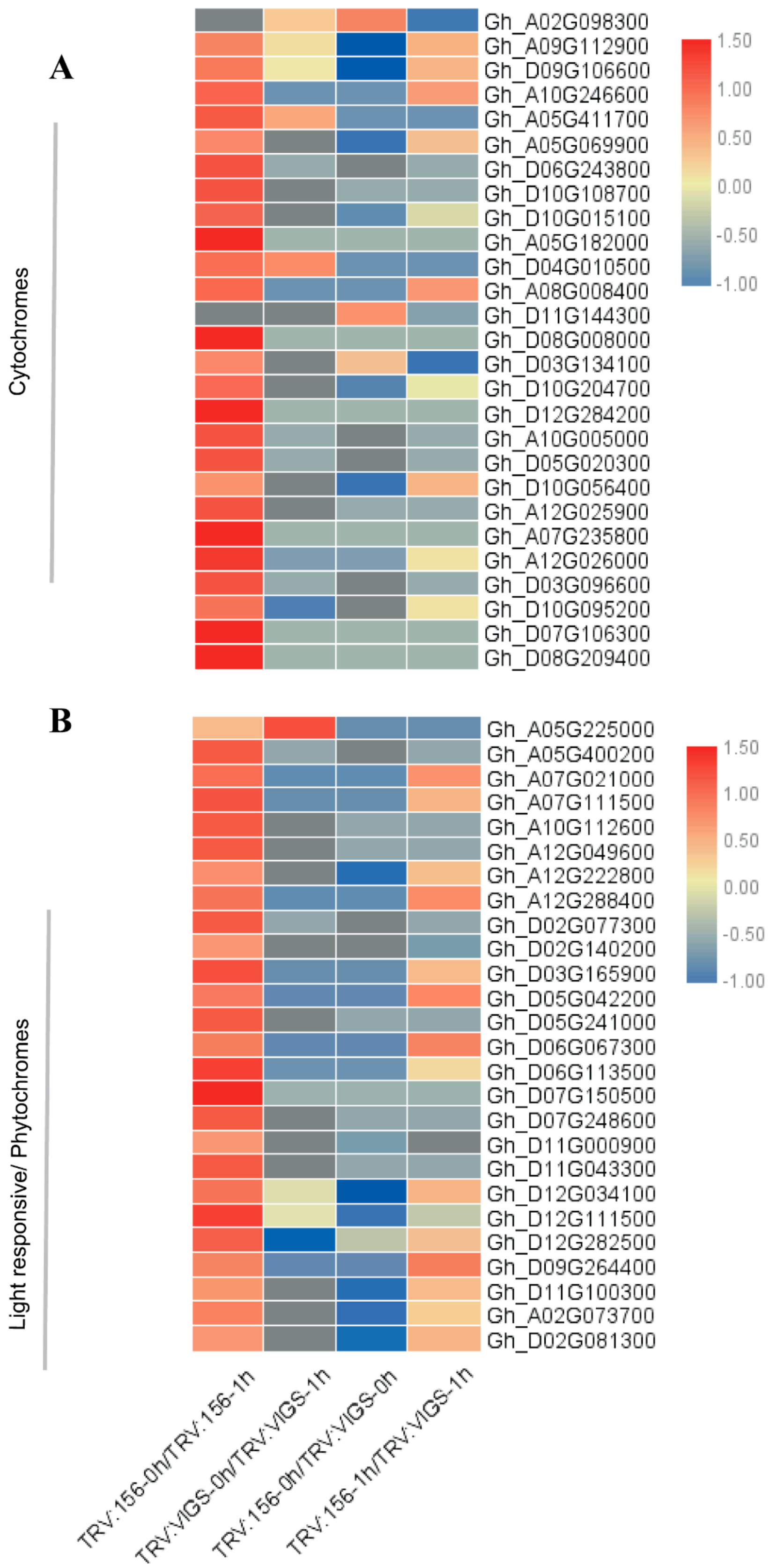
A
Cytochromes
Gh_A02G098300
Gh_A09G112900
Gh_D09G106600
Gh_A10G246600
Gh_A05G411700
Gh_A05G069900
Gh_D06G243800
Gh_D10G108700
Gh_D10G015100
Gh_A05G182000
Gh_D04G010500
Gh_A08G008400
Gh_D11G144300
Gh_D08G008000
Gh_D03G134100
Gh_D10G204700
Gh_D12G284200
Gh_A10G005000
Gh_D05G020300
Gh_D10G056400
Gh_A12G025900
Gh_A07G235800
Gh_A12G026000
Gh_D03G096600
Gh_D10G095200
Gh_D07G106300
Gh_D08G209400
1.50
1.00
0.50
0.00
-0.50
-1.00
B
Light responsive/ Phytochromes
Gh_A05G225000
Gh_A05G400200
Gh_A07G021000
Gh_A07G111500
Gh_A10G112600
Gh_A12G049600
Gh_A12G222800
Gh_A12G288400
Gh_D02G077300
Gh_D02G140200
Gh_D03G165900
Gh_D05G042200
Gh_D05G241000
Gh_D06G067300
Gh_D06G113500
Gh_D07G150500
Gh_D07G248600
Gh_D11G000900
Gh_D11G043300
Gh_D12G034100
Gh_D12G111500
Gh_D12G282500
Gh_D09G264400
Gh_D11G100300
Gh_A02G073700
Gh_D02G081300
1.50
1.00
0.50
0.00
-0.50
-1.00
TRV:156-0h/TRV:156-1h
TRV:VIGS-0h/TRV:VIGS-1h
TRV:156-0h/TRV:VIGS-0h
TRV:156-1h/TRV:VIGS-1h

**Supplementary Figure S2. Phtotoreceptors and Light responsive genes expression**. Differentially expressed DEGs in TRV:156-0h/TRV:156-1h (a) Cytochrome related genes (b) Phytochromes and other light responsive genes. DEGs details is provided in (Supplementary File 1)
