# Supplementary material for: Transcriptome Analysis Revealed GhWOX4 Intercedes Myriad Regulatory Pathways to Modulate Drought Tolerance and Vascular Growth in Cotton
Source: Int J Mol Sci. 2021 Jan 18;22(2):898. doi: 10.3390/ijms22020898 (PMC7829754; doi:10.3390/ijms22020898)
Supplement: Supplementary file 1 [file ijms-22-00898-s001.zip › Table S1. Promotor CAREs details.pdf]

Table S1. Identified cis-regulatory elements and associated functions in the promotor region of GhWOX4.

Motif	Sequence	Function	Element Abundance
AT1-motif	AATTATTTTTTATT	part of a light responsive module	1
LTR	CCGAAA	cis-acting element involved in low-temperature responsiveness	1
MYB	TAACCA	Dehydration and stress responses	1
chs-CMA1a	TTACTTAA	part of a light responsive element	1
STRE	AGGGG	Common in transcriptional activation and stress-responsive genes	1
GT1-motif	GGTTAA	light responsive element	1
O2-site	GATGA(C/T)(A/G)TG(A/G)	cis-acting regulatory element involved in zein metabolism regulation	2
AAGAA-motif	gGTAAAGAAA	Unknown	1
MYB-like sequence	TAACCA	Dehydration and stress responses	1
Box 4	ATTAAT	part of a conserved DNA module involved in light responsiveness	9
WUN-motif	AAATTACT	Wound and MeJA-responsive motif	1
MYC	CAATTG	promote cell proliferation/growth, dehydration & Chilling Response	5
ERE	ATTTTAAA	Ethylene responsive might involve in signaling and defense	8
ARE	AAACCA	cis-acting regulatory element essential for the anaerobic induction	1
TCCC-motif	TCTCCCT	part of a light responsive element	1
I-box	atGATAAGGTC	part of a light responsive element	1
Myc	TCTCTTA		2
CAAT-box	CAAT	Common cis-acting element in promoter and enhancer regions	35
