# Supplementary material for: Transcriptome Analysis Revealed GhWOX4 Intercedes Myriad Regulatory Pathways to Modulate Drought Tolerance and Vascular Growth in Cotton
Source: Int J Mol Sci. 2021 Jan 18;22(2):898. doi: 10.3390/ijms22020898 (PMC7829754; doi:10.3390/ijms22020898)
Supplement: Supplementary file 1 [file ijms-22-00898-s001.zip › Table S2. Primers used in study.pdf]

Primers sequences used in present study.

GhWOX4:

GhWOX4 Forward Primer (5'-3'):
CAAAAAAGCAGGCTCAGGGGATATCATGGGAAACATGAAGATGCAC

GhWOX4 Reverse Primer (5'-3'):

CCATTGCACCCGGAAAGCAGAGATATCGCCCTGCACCCAGCTTT

VIGS_GhWOX4 Forward Primer (5'-3'):
AAGGTTACCGAATTCTCTAGAGCTCTGCTCCCATTACTCCAAT

VIGS_GhWOX4 Reverse Primer (5'-3'):

TTGCACCCGGAAAGCAGATGAGGATCCGGTACCGAGCTC

qGhWOX4-F: GAAGGCAAAAACGTGTTC

qGhWOX4-R: CCAAAGTTATTGGAGTAATG

AtActin-F: GTCGTACAACCGGTATTGTGC

AtActin-R: CACAAACGAGGGCTGGAACAAG

GhActin-F: ACTCTCCCGCTATGTATGTCGC

GhActin-R: AGAAACCCTCGTAGATTGGCAC
